# Baseline azithromycin resistance in the gut microbiota of preterm born infants

**DOI:** 10.1038/s41390-023-02743-7

**Published:** 2023-08-07

**Authors:** David J. Gallacher, Lei Zhang, Ali F. Aboklaish, Emma Mitchell, Richard Wach, Julian R. Marchesi, Sailesh Kotecha

**Affiliations:** 1 Neonatal Unit, University Hospital of Wales, Cardiff, UK; 2 Department of Child Health, Cardiff University School of Medicine, Cardiff, UK; 3 Neonatal Unit, Southmead Hospital, Bristol, UK; 4 Division of Digestive Diseases, Faculty of Medicine, Imperial College London, London, UK

## Abstract

**Background:**

Macrolides, including azithromycin, are increasingly used in preterm-born infants to treat *Ureaplasma* infections. The baseline carriage of macrolide resistance genes in the preterm stool microbiota is unknown.

**Objectives:**

Identify carriage of azithromycin resistant bacteria and the incidence of macrolide resistant genes.

**Methods:**

Azithromycin resistant bacteria were isolated from serial stool samples obtained from preterm infants (≤32 weeks’ gestation) by culturing aerobically/anaerobically, in the presence/absence of azithromycin. Using quantitative PCR, we targeted 6 common macrolide resistance genes (*erm(A)*, *erm(B)*, *erm(C)*, *erm(F)*, *mef(A/E), msr(A)*) in DNA extracted from selected bacteria resistant to azithromycin.

**Results:**

From 89 stool samples from 37 preterm-born infants, 93.3% showed bacterial growth in aerobic or anaerobic conditions. From the 280 azithromycin resistant isolates that were identified, *Staphylococcus* (75%) and *Enterococcus* (15%) species dominated. Macrolide resistance genes were identified in 91% of resistant isolates: commonest were *erm(C)* (46% of isolates) and *msr(A)* (40%). Multiple macrolide resistance genes were identified in 18% of isolates.

**Conclusion:**

Macrolide resistance is common in the gut microbiota of preterm-born infants early in life, most likely acquired from exposure to the maternal microbiota. It will be important to assess modulation of macrolide resistance, if macrolide treatment becomes routine in the management of preterm infants.

**Impact Statement:**

- Azithromycin resistance is present in the stool microbiota in the first month of life in preterm infants
- 91% of azithromycin resistant bacteria carried at least one of 6 common macrolide resistant genes
- Increasing use of macrolides in the preterm population makes this an important area of study

## Introduction

The use of macrolides, commonly azithromycin, is increasing in preterm-born infants especially to treat *Ureaplasma* species^1^ to prevent the development of chronic lung disease of prematurity (CLD, also called bronchopulmonary dysplasia, BPD).^2,3^ The immunomodulatory effects of azithromycin is also likely to target pulmonary inflammation, an important contributory factor to the development of CLD.^4^ The AZTEC (Azithromycin therapy for chronic lung disease of prematurity) trial is currently evaluating if early treatment with azithromycin of infants born at <30 weeks’ gestation improves survival without development of CLD^5^ and respiratory disease in infancy.^6^ If successful, prescription of macrolide antibiotics in preterm infants is likely to increase significantly.

Antibiotic resistance is an emerging worldwide issue and the widespread use of macrolide antibiotics is associated with increased carriage of multiple antibiotic resistance genes.^7^ The effect of azithromycin on the carriage of macrolide resistance genes in preterm infants is unknown. However, in adult patients with COPD and cystic fibrosis, long term treatment with azithromycin increases prevalence of macrolide resistance.^8,9^ Multi-resistant bacteria causing infections in neonatal patients are a significant issue^10^ likely to be associated with increased morbidity and mortality.

The neonatal gut microbiota is thought to be acquired during labour and postnatally, and is significantly affected by mode of delivery,^11,12^ with organisms acquired from both the maternal microbiota and from the local environment.^13^ The preterm gut microbiota is a dynamically changing community of microorganisms influenced by feeding practices; use of antibiotics and probiotics; and by other environmental factors.^12^ The gut microbiota is a known reservoir of antibiotic resistance genes,^14^ and understanding the effects of antibiotics on commensal organisms is as important as monitoring resistance in invasive pathogens in developing antibiotic stewardship strategies.^15^

Antibiotic resistance genes have previously been detected in significant numbers in the gut microbiota of term infants^16–18^ with vertical transmission of antibiotic resistant organisms being hypothesised although antibiotic resistant organisms have also been detected in human breast milk as another potential source for acquisition.^18,19^ However, evidence from preterm infants is lacking. A previous study of preterm infants used metagenomic sequencing to detect antibiotic resistance genes to many classes of antibiotics in the gut microbiota. The study identified presence of genes conferring the macrolide-lincosamide-streptogramin B resistance phenotype, which resulted in azithromycin resistance in infants born at ≤32 weeks’ gestational age, but organisms carrying the antibiotic resistant genes were not reported.^20^ Another study highlighted use of β-lactam antibiotics increasing carriage of antibiotic resistance genes.^21^

In this study, we aimed to identify baseline carriage of macrolide resistance genes in the microbes identified in stool samples from preterm infants ≤32 weeks gestational age at birth, who were at risk of developing CLD. Identification of the specific bacteria carrying azithromycin resistance genes should not only aid the understanding of the potential sources of macrolide resistant bacteria in preterm infants, but also the potential pathogenic potential for disease including sepsis in vulnerable preterm-born infants.

## Materials and methods

### Sample collection and processing

Stool samples were collected from preterm infants (≤32 weeks’ gestational age at birth) recruited for an observational study of the neonatal microbiota from two tertiary neonatal units in the UK.^12^ Written informed consent was provided by parents with agreement for storage and further testing of samples. Ethics approval was granted by the Wales Research Ethics Committee 2 (Reference: 14/WA/0190). Methods of sample collection and storage have been previously described.^12^ In brief, stool samples were collected weekly during the first week of life in preterm infants who required ventilation for respiratory distress and aliquoted prior to freezing at −80 °C.

### Culture and identification of azithromycin resistant bacteria

Faecal slurry was made by diluting 20–60 mg of defrosted stool in 500 µL of 1X sterile phosphate buffered saline (PBS). Serial dilutions (10^0^ to 10^−5^) of faecal slurry were prepared using sterile PBS. The serial dilutions of faecal slurry from each sample was cultured on YCFA media^22^ with and without azithromycin supplementation in aerobic and anaerobic conditions, using drop counts to enable semi-quantification of bacterial growth. YCFA media has been shown to be one of the most appropriate media for culturing gut bacteria.^23^ A previous study had reported MIC_90_ for azithromycin of 2 µg/ml and 8 µg/ml for *Staphylococcus* and *Escherichia coli* respectively thus we obtained drop counts (3 × 10 µl) for bacterial growth resulting after 24 h of growth on YCFA media with and without 8 µg/ml of Azithromycin^24^ in aerobic and anaerobic conditions. The percentage of azithromycin resistant bacteria was calculated from these count results. Up to two azithromycin resistant colonies from each azithromycin containing plate per sample were selected and further sub-cultured in YCFA broth. DNA was extracted from azithromycin resistant colonies using a bead beating process combined with the Maxwell 16 Cell DNA purification kit. Near complete sequencing of the 16S rRNA gene was performed for species identification. 16S rRNA gene amplification was performed using universal primers (sequences listed in Supplementary Table S2) and the successfully amplified PCR products underwent Sangar sequencing (Eurofins Genomics, Ebersberg, Germany). Species identification was made via the NCBI BLAST database, and alignment with known gene sequences. 16S rRNA gene sequences of >97% similarity and E value of 1 × 10^−5^ were used to confirm species identification, if multiple species met the criteria a majority rule was employed.

### Identification of azithromycin resistance genes

Azithromycin resistance genes within the bacterial genome of all the cultured azithromycin resistant isolates were identified by quantitative PCR (qPCR) assay. The assay was designed to identify the presence of six of the most common macrolide resistance genes (*erm(A)*, *erm(B)*, *erm(C)*, *erm(F), mef(A/E)* and *msr(A)*). Two reference plasmids were designed and synthesised, each containing 3 macrolide resistance genes. Reference sequences for the macrolide resistance genes used for primer/probe design and for use in the reference plasmids were taken from the Comprehensive Antibiotic Resistance Database.^25^ Primer and probe sequences were designed by the online web interface for Primer3,^26^ except for those used for the *erm(B)* gene which were based on a published protocol.^27^ Two separate multiplex qPCR reactions were performed with primers and TaqMan probes listed in Supplementary Table S3 to amplify each resistance gene. The qPCR was performed in a final volume of 10 µl reaction mixture, containing 5 µl of 2X SsoAdvanced Universal Probes Supermix (Bio-Rad Laboratories Ltd, UK), 1.4 µl DNase/RNase-Free Distilled Water (Severn Biotech Ltd, UK), 0.5 µl of each primer (concentration 10 µM), 0.2 µl probes and 2.5 µl DNA template. All qPCR reactions were performed in a CFX96 Touch Real-Time PCR detection system (Bio-Rad Laboratories Ltd, UK) using the following conditions: initial incubation at 95 °C for 5 min, followed by 40 cycles for 15 s at 95 °C and 30 s at 60 °C. A standard curve of 1:10 serially diluted concentrations of the standard DNA plasmid (copy numbers ranging from 1 to 10^6^), was produced for each run of the assays. Samples and controls were run in duplicate with those finding >100 copies/reaction considered to contain the macrolide resistance gene. Results between duplicates for the *erm(F)* gene were inconsistent thus were repeated in single qPCR reactions resulting in consistent results between duplicate reactions. PCR products from examples of each successfully amplified macrolide resistance gene underwent Sangar sequencing (Eurofins Genomics, Ebersberg, Germany) to confirm amplification of the correct sequence.

### Stool microbiota identification

The stool microbiota data for the complete cohort has been previously published including the detailed methodology.^12^ The data for the relevant samples has been included in this study to relate the azithromycin resistant organisms to the whole bacterial community. The methodology of 16S rRNA gene sequencing is therefore summarised: 10% w/v faecal slurry was produced from 200 mg of stool and DNA extraction was performed using the Qiagen Stool Mini Kit, combined with a bead beating step, from a cell pellet produced from centrifugation of the faecal slurry. 16S rRNA gene paired end sequencing was performed using barcoded primers for the V3-V4 hypervariable region of the 16S rRNA gene (Supplementary Table S1) using an Illumina MiSeq device. Data analysis and establishing operational taxonomic units (OTU) using a 97% similarity threshold (opticlust method) with taxonomic assignment to genus level made by aligning sequences to the Silva Database (version 136)^28^ and comparing representative sequences to the Ribosomal Database Project reference library (release 11.5)^29^ using Mothur v1.39.5.^30^ Statistical analysis was performed in R v4.2.2^31^ using non-parametric tests to compare groups. *p* value < 0.05 was taken to be statistically significant.

## Results

A total of 89 remaining stool samples from the previous study^12^ from 37 (67.3% of the original cohort) preterm infants (mean 2.4 samples per infant) were cultured to detect the presence of azithromycin resistant organisms. The demographic details of recruited infants are shown in Table 1. The recruited infants had a median gestational age at birth of 26^+0^ (IQR: 24^+5^, 28^+1^) weeks and a median birth weight of 820 g (IQR: 690 g, 940 g). According to local policy, all infants were fed either mother’s or donor breast milk except for two infants who received formula milk during week 4 of life. Six infants had received macrolide antibiotics during the first 28 days of life, (1x clarithromycin, 5x erythromycin) for confirmed *Ureaplasma* infection. Of these, 2 infants only had stool samples collected prior to receiving the macrolide antibiotic. Nine samples (10.1%) from 4 infants were collected after exposure to macrolide antibiotics. Of the 89 total samples, 80 samples (90%) demonstrated bacterial growth on YCFA plates under aerobic conditions, and 80 samples showed bacterial growth under anaerobic conditions, with 6 samples not demonstrating bacterial growth in either condition.

**Table 1:** Demographic details of recruited preterm infants.

Number of preterm neonates	37
Sex, Male (*n*,%)	24 (64.9%)
Gestation (median, IQR)	26^+0^ weeks (24^+5^, 28^+1^)
Birth Weight (median, IQR)	820 g (690, 940)
Maternal Age (mean, St Dev)	29.5 years (6.0)
Multiple Pregnancy (*n*, %)	11 (29.7%)
Complete Course of Antenatal Steroids (*n*, %)	22 (59.5%)
Delivery mode, Vaginal (*n*, %)	22 (59.5%)
Recruitment Site^a^ (Cardiff: Bristol) (*n*, %)	14:23 (37.8%:62.2%)
Length of neonatal admission (median, IQR)	92 days (66–118)
NEC (Bells stage ≥2) (*n*, %)	5 (13.5%)
Chronic Lung Disease (O_2_ at 36 weeks) (*n*, %)	29 (78.4%)

*NEC* Necrotising Enterocolitis

^a^Samples were collected from 2 recruitment sites in Cardiff and Bristol

**Fig. 1:**
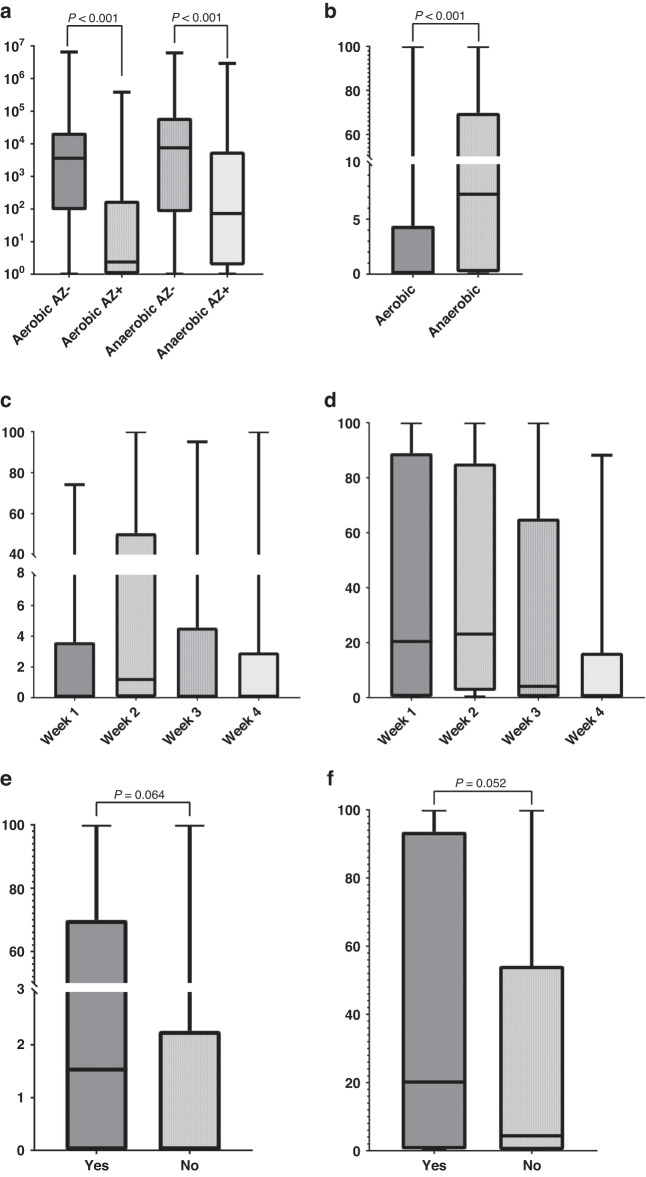
Azithromycin resistant organisms within stool samples of preterm infants. **a** Box plot of growth of colony forming units of bacteria on YCFA plates with (Az + ) and without (Az-) azithromycin supplementation (8 µg/ml) comparing growth in aerobic and anaerobic conditions (*N* = 89). **b** Percentage of colony forming units demonstrating azithromycin resistance in all samples comparing growth in aerobic and anaerobic conditions. **c** Box plot showing percentage resistance to azithromycin of colony forming units demonstrating azithromycin resistance when cultivated under aerobic conditions during the first 4 weeks of life. Week 1 *n* = 22, Week 2 *n* = 17, Week 3 *n* = 22, week 4 *n* = 28. **d** Box plot showing percentage resistance to azithromycin of colony forming units demonstrating azithromycin resistance when cultivated under anaerobic conditions during the first 4 weeks of life. Week 1 *n* = 22, Week 2 *n* = 17, Week 3 *n* = 22, week 4 *n* = 28. **e** Box plot comparing percentage resistance to azithromycin of colony forming units in samples collected when infant receiving intravenous antibiotics compared to samples collected when infant not receiving intravenous antibiotics when grown in anaerobic conditions. No *n* = 54, Yes *n* = 35. **f** Box plot comparing percentage resistance to azithromycin of colony forming units in samples collected when infant receiving intravenous antibiotics compared to samples collected when infant not receiving intravenous antibiotics when grown in anaerobic conditions. No *n* = 54, Yes *n* = 35.

Figure 1a shows the drop count data used to measure the colony forming units (cfu) of bacteria in faecal slurry from clinical samples. The results demonstrated that significantly fewer colonies were cultured on YCFA plates supplemented with azithromycin compared to the no azithromycin plate for both aerobic and anaerobic conditions. Thus, the azithromycin concentration used was sufficient to suppress bacterial growth of susceptible organisms. All clinical samples which showed no bacterial growth on plates without Azithromycin supplementations, also showed no growth under the same conditions when cultured in the presence of Azithromycin. Figure 1b shows that a median of 0.75% of colonies demonstrated azithromycin resistance in aerobic conditions compared to a median of 7.3% in anaerobic conditions. Figure 1c shows that the median percentage of cfu demonstrating azithromycin resistance when grown in aerobic conditions did not change over time (*p* = 0.263); however, when grown in anaerobic conditions resistance decreased during the first 4 weeks of life from a median of 20.5% of colony forming units in the first week of life to 0.8% in the 4^th^ week of life (Fig. 1d) (*p* = 0.012). The range of percentage resistance when grown in both aerobic and anaerobic conditions was large at each time point. Figure 1e, f demonstrate a trend towards increased azithromycin resistance in stool samples that were taken while the infant was receiving intravenous antibiotic treatment compared to samples collected when not on antibiotics in both aerobic and anaerobic conditions. One recruiting site used a probiotic containing *Lactobacillus acidophilus* and *Bifidobacterium infantis* routinely for preterm infants during first month of life. Supplementary data Fig. 1 shows there was no effect of probiotics on azithromycin resistance.

From samples with identifiable colonies, up to two azithromycin resistant colonies were picked from each plate cultured in aerobic and anaerobic conditions for additional taxonomic characterisation. Colonies were picked based on their prominence and morphology to identify at two different species if possible. A total of 122 colonies grown under aerobic conditions and 158 colonies grown under anaerobic conditions were selected. Table 2 demonstrates the genus level identification of these resistant isolates over time. The data show that the percentage of infants colonised with azithromycin resistant *Staphylococcus* remained static in aerobic conditions but decreased from 80% to 60.9% in anaerobic conditions. In contrast, *Enterococcus* increased in both aerobic and anaerobic conditions during the first four weeks of life.

**Table 2:** Table demonstrating number and percentage of infants with available stool samples at each week with identified azithromycin resistant organisms by genus.

	Week 1 *N* = 20	Week 2 *N* = 16	Week 3 *N* = 17	Week 4 *N* = 23	At any timepoint
**Aerobic**					
*Staphylococcus*	10 (50.0)	13 (81.2)	8 (47.1)	12 (52.2)	28 (75.6)
*Enterococcus*	0 (0.0)	1 (6.3)	2 (11.7)	5 (21.7)	7 (18.9)
Other	0 (0.0)	0 (0.0)	0 (0.0)	1 (4.3)	1 (2.7)
Undetermined/No Result	0 (0.0)	1 (6.3)	1 (5.9)	3 (13.0)	4 (10.8)
Total	10 (50.0)	15 (93.8)	10 (58.8)	21 (91.3)	35 (94.6)
**Anaerobic**					
*Staphylococcus*	16 (80.0)	12 (75.0)	12 (70.6)	14 (60.9)	31 (83.7)
*Enterococcus*	1 (5.0)	1 (6.3)	2 (11.6)	8 (34.8)	12 (32.4)
Other	0 (0.0)	1 (6.3)	1 (5.9)	0 (0.0)	2 (5.4)
Undetermined/No Result	0 (0.0)	0 (0.0)	0 (0.0)	0 (0.0)	0 (0.0)
Total	16 (80.0)	14 (87.5)	14 (82.4)	21 (91.3)	35 (94.6)

Some infants had organisms from 2 genera detected at some timepoints.

**Fig. 2:**
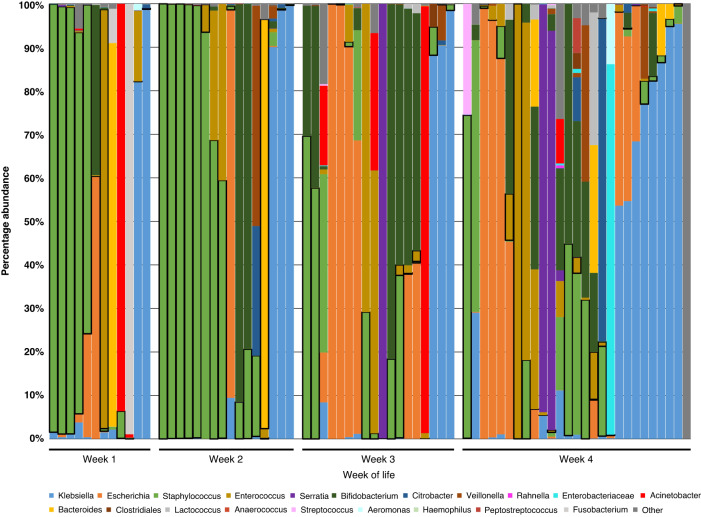
Relative abundance at genus level of bacteria within stool samples detected by sequencing the V3-V4 region of the bacterial 16S rRNA gene, split by week of life. The most abundant 20 genera shown (98.8% of reads). Boxes around specific genera demonstrate the genus from which azithromycin resistant organisms in the sample were from.

Figure 2 demonstrates the relative abundance of organisms in the stool microbiota of the samples tested and how the azithromycin resistant isolates relate to the overall gut microbiota. Of the 89 samples, 72 (80%) had successful 16S rRNA gene sequencing data available to demonstrate the composition of the stool microbiota. The figure shows our previously published finding of a decrease in the proportion of *Staphylococcus* species during the first 4 weeks of life and an increase in the proportion of Gram-negative genera over time.^12^ Figure 2 and Table 2 demonstrate the high proportion of *Staphylococcus* species that were cultured from stool samples with 75% of all isolates from this genus. The next most identified organisms were from the *Enterococcus* genus (15%). Figure 2 demonstrates that in some samples, the organisms cultured were from a genus that dominated the microbiota; however, for some samples, the cultured organism was from a genus that made up only a small proportion of the infant’s overall gut microbiota.

**Fig. 3:**
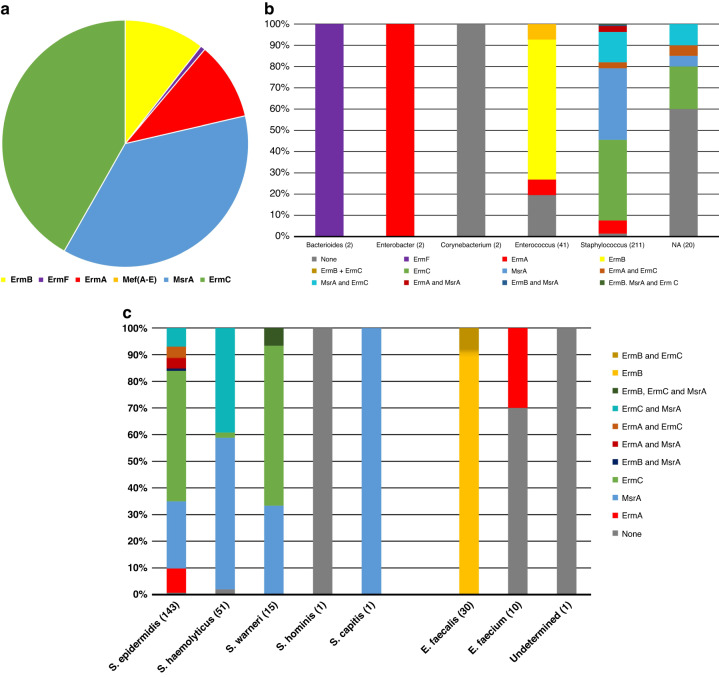
Macrolide resistance genes identified within azithromycin resistant isolates culture from the stool of preterm infants. **a** Pie chart demonstrating the relative abundance of each of the 6 macrolide resistance genes tested for using qPCR methodology. Total number of genes identified = 354. **b** Macrolide resistance genes identified by genus of azithromycin resistant isolate. **c** Macrolide resistant genes identified within the *Staphylococcus* and *Enterococcus* genera by species of bacteria.

Of the 280 bacteria isolated with azithromycin resistance, 278 (99.3%) were tested for the presence of the six common macrolide resistance genes. Figure 3a shows the macrolide resistance genes identified in azithromycin resistant bacteria. The most identified gene was *erm(C)* (45.6% of isolates) followed by *msr(A)* (40.2% of isolates). Of the 6 genes that were tested for, only one (m*ef(A/E)*) was not detected in any of the isolates. Of the 278 isolates, at least one macrolide resistance gene explaining the resistance to azithromycin was detected in 253 (91.0%) of the isolates. The presence of two different macrolide resistance genes was identified in 49 isolates (17.6%) and three different genes were identified in 1 isolate (0.4%).

Figure 3b shows the breakdown of macrolide resistance genes identified by genus of the isolates. *erm(F)* was only identified in isolates from the *Bacteroides* genus. Both *Enterobacter* isolates, from the same infant, contained only the *erm(A)* gene. In 73.2% of the *Enterococcus* isolates, the *erm(B)* gene was identified, with *erm(B)* only being identified in two isolates that were not from the *Enterococcus* genus. *Staphylococcus* isolates had the widest variety of identified macrolide resistance genes; however, 85.8% of these isolates contained either the *msr(A)* or *erm(C)* gene, either alone or in combination.

Figure 3c shows further breakdown of antibiotic resistance genes identified by species within the two commonest genera, *Staphylococcus* and *Enterococcus*. Within *Staphylococcus*, the *erm(A)* gene was only identified in *S. epidermidis*, while *msr(A)* and *erm(C)* dominated each of the three most identified species: *S. epidermidis*, *S. haemolyticus* and *S. warneri*.

Two of the 9 samples collected after exposure to macrolide antibiotics failed to show any bacterial growth in aerobic or anaerobic conditions. Bacterial resistance to azithromycin was more prevalent in stool samples exposed to azithromycin than in other samples. Median azithromycin resistance in aerobic conditions was 27.5% for stool samples from infants exposed to macrolides compared to 0.03% for stool samples from unexposed infants (*p* = 0.04). In anaerobic conditions the corresponding percentages were 85.1% and 6.0% respectively (*p* = 0.054). A total of 26 azithromycin resistant bacterial colonies were identified from the samples collected after exposure to azithromycin, with 23 of these being *Staphylococcus* species. From the cultured bacteria, a single macrolide resistance gene was identified in 24 organisms (*msr(A)* = 8 organisms, *erm(C)* = 16 organisms).

## Discussion

To our knowledge, this is the first study specifically assessing the prevalence of macrolide resistance genes within the gut microbiota of preterm infants. This study of infants who were predominantly not receiving macrolides, establishes that azithromycin resistant organisms are identifiable in the majority of stool samples obtained from preterm infants in the first 4 weeks of life. Overall, the carriage of azithromycin organisms in the stool microbiota appeared to decrease over time. This carriage was most likely to be due to a decrease in the *Staphylococcus* species within the gut microbiota seen during the first month of life, as previously reported.^12^

The most commonly identified azithromycin resistant organisms were from the *Staphylococcus* genus, known to dominate the early microbiota in many preterm infants, particularly those born by caesarean section.^11,32^ The dominant azithromycin resistance genes identified were *msr(A)* and *erm(C)*, genes known to be commonly associated with the *Staphylococcus* genus.^25^ However, 5 out of the 6 macrolide resistance genes tested for were identified in the azithromycin resistant isolates, from 5 different bacterial genera examined, showing that macrolide resistance is present across a range of bacterial taxa, with a range of different genes contributing to the resistance pattern. Azithromycin resistance was detected in bacteria from stool samples collected during the first week of life, supporting the hypothesis that vertical transmission of maternal microbiome together with antibiotic resistance genes seems to be occurring during labour.^18^ The mechanisms of such transfer is not fully clear, but the mode of delivery studied mainly in term born infants has been shown to influence the resulting stool microbiota in the infant with skin associated staphylococci being associated more with caesarean sections and vaginal bacteria, lactobacilli, and some Gram negative bacteria more with vaginal delivery. Less is known about preterm born infants who may not be colonised by the maternal bacterial community as often they are admitted directly to neonatal units, especially those who require immediate respiratory support.^31^ Our data led us to conclude that increased proportion of infants had detectable azithromycin resistant genes during the first 4 weeks of life, however the proportion of resistant bacteria within the samples decreased over this time, when grown in anaerobic conditions. This may suggest that there is ongoing acquisition of azithromycin resistant bacteria during the first month of life or more likely selection of azithromycin resistance bacteria as sensitive ones will be removed. Alternatively, the increasing proportion of infants with azithromycin resistant *Enterococcus* species during the first 4 weeks of life may also reflect postnatal acquisition. Gene transfer between organisms in the infant gut could also explain the increasing proportion of infants colonised with azithromycin resistant *Enterococcus* species during the first month of life. All the infants included in this study were preterm infants requiring mechanical ventilation and received postnatal antibiotics.

Macrolide resistance is conferred by different mechanisms for the genes we have studied. Expression of the *Erm* family of genes results in resistance to macrolides, lincosamides, and streptogramin B, the MLS_B_ phenotype due to methylation of the ribosomal target of the antibiotics.^33^ The *Erm* genes were detected in bacteria known to carry these genes according to the published literature (*erm(F)* in *Bacteroides*, *erm(B)* in enterococci, *erm(A)* in staphylococci and enterococci).^25^ Both the *msr(A)* gene and the *mef(A/E)* gene confer resistance via different efflux pumps.

Many macrolide resistance genes are located on plasmids or other mobile genetic elements in the genome of microorganisms.^34^ With transfer of antibiotic resistant genes between bacteria within the gut environment a documented phenomenon,^35^ including the infant gut,^36^ understanding antibiotic resistance genes in the neonatal gut is an important area for further study. This study establishes an understanding of the baseline of macrolide resistance in preterm infants, without widespread use of azithromycin. The effect of routine azithromycin therapy to preterm infants on carriage of azithromycin resistance genes remains to be established.

Previous studies of preterm infants have suggested that antibiotic resistance genes in stool samples for a specific class of antibiotic are enriched following treatment with that antibiotic, often contributed to by a single species^37^ however, macrolide antibiotics have not been studied thus far. Efforts to reduce the carriage of antibiotic resistance genes in breastfed term infants using probiotics have shown significantly less antibiotic resistance in infants taking the probiotic supplement compared to controls.^38^ Our study did not show any effects of probiotic use. However, this strategy may still be useful in trying to reduce overall antibiotic resistance gene carriage in the preterm-born population and requires further robust investigation in trials of probiotic preparations in this population. The benefits of trying to minimise the presence of antibiotic resistance genes in the preterm neonatal gut may go beyond the neonatal period as multidrug resistant bacteria and the overall carriage of antibiotic resistance genes persists during the first months of life in ex-preterm infants.^39^

This study has a number of limitations. Stool samples were frozen −80 °C before the culture process thus may have affected the culture yield of the more fragile organisms, but is unlikely to affect the antibiotic resistance genes identified. YCFA media was chosen as it attempts to mimic the gut environment and is widely used in studies of the gut microbiota.^40^ Any culture media and culturing methods may impact the ability of certain organisms to grow, and this may have encouraged the growth of the *Staphylococcus* species which dominated our results, however, YCFA has been shown to be one of the most appropriate media for culture of gut bacteria.^23^

Maternal use of macrolides before delivery was not recorded. It is likely that some of the mothers may have received oral erythromycin prior to delivery as it is often used in mothers who have a history of prelabour rupture of membranes. Nine samples were collected after the infant had received a macrolide antibiotic. The small number of samples taken after macrolide exposure limits the interpretation of the results comparing those samples taken with and without macrolide exposure, however, does suggest the impact of macrolide exposure on antibiotic resistance requires further investigation. The small proportion of samples taken after exposure to macrolide antibiotics is unlikely to have affected the overall results.

The subjective picking of prominent colonies for further investigation may have contributed to the dominance of *Staphylococcus* species within the azithromycin resistant isolates, as these are known to grow in more prominent colonies than other bacteria. Other genes than the 6 investigated in this study may contribute to azithromycin resistance, but these 6 genes accounted for 91% of the resistance to azithromycin, so other genes are less likely to contribute significantly.

Despite these limitations, we have reported the macrolide genes associated with azithromycin resistance in bacteria isolated from the preterm gut. We have also provided validated qPCR methodology to identify presence of macrolide resistance genes in the DNA obtained from stool samples from preterm-born infants. The organisms cultured were bacteria well known to be present in the stool of preterm infants. The presence of antibiotic resistance genes within a genome does not equate to expression and functional antibiotic resistance, however each of the isolates in this study has been shown to demonstrate azithromycin resistance, increasing the likelihood that the genes identified were conferring antibiotic resistance in these bacteria. Since azithromycin is increasingly used in preterm-born infants, it will be importantly to investigate what effect increased use of azithromycin on the macrolide resistance genes over time.

### Supplementary information

Supplementary Material

## Data Availability

16S rRNA gene sequencing data for the microbiota analysis has been uploaded to the European Nucleotide Archive, accession number PRJEB61860. The remaining dataset are available on request from the corresponding author on reasonable request.
