# Supplementary material for: Baseline azithromycin resistance in the gut microbiota of preterm born infants
Source: Pediatr Res. 2023 Aug 7;95(1):205–12. doi: 10.1038/s41390-023-02743-7 (PMC10798878; doi:10.1038/s41390-023-02743-7)
Supplement: Supplementary file 1 — Supplementary Material [file 41390_2023_2743_MOESM1_ESM.pdf]

Supplementary Figure 1 for online only

Azithromycin Resistance by Probiotic Use

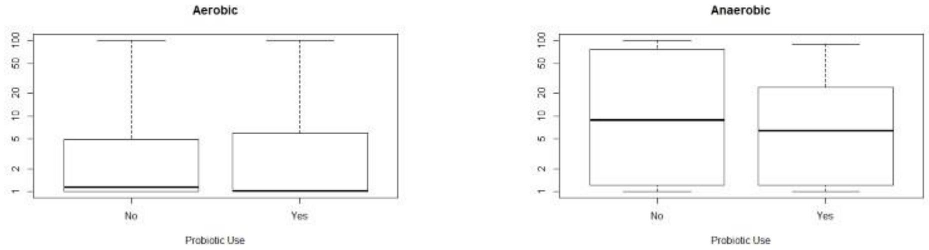

p=0.744

p=0.288

Supplementary Figure 1: Box plots comparing percentage azithromycin resistance between samples taken while the infant was a receiving a probiotic preparation and samples taken when not on a probiotic preparation. (Yes n=25, No n= 64)

Supplementary Online Data Tables

Table S1: Primers used for sequencing V3-V4 region of the 16S rRNA gene for microbiome analysis

Primer	Sequence (5'-3')
Forward	TCCTACGGGAGGCAGCAGT
Reverse	GGACTACCAGGGTATCTAATCCTGTT

Table S2: Primers used for PCR amplification of 16S gene for species identification of azithromycin resistant isolates from stool.

Primer	Sequence (5'-3')
Forward (27F)	AGAGTTTGATCMTGGCTCAG
Reverse (1492R)	GGTTACCTTGTTACGACTT

Table S3: Primer and Probe Sequences used for macrolide resistance qPCR assay

Gene	Primer/Probe	Sequence (5'-3')
erm(B)	Forward	CTTGGATATTCACCGAACAC
	Reverse	TTGGTTTAGGATGAAAGCAT
	Probe	[FAM]AAGTCTCGATTCAGCAATTGCTTAAG[BHQ1]
erm(F)	Forward	GCCCGAAATGTTCAAGTTGT
	Reverse	CAATGGAACCTCCCAGAAAA
	Probe	[HEX]TTTGCAGTTCCGAAATTTCC[BHQ1]
erm(A)	Forward	GCAACGAGCTTTGGGTTTAC
	Reverse	TCAATGGTTGATGTCGTTCAA
	Probe	[ROX]TGGAGATGGATATAAAAATGCTCA[BHQ2]
mef(A/E)	Forward	TGCGCAGGCTATAGTCAGTCT
	Reverse	CAATCACAGCACCCAATACG
	Probe	[FAM]ATTGTTAGTCCGGCAGTTGC[BHQ1]
msr(A)	Forward	CAAATGGCACAAGCATCATC
	Reverse	TGCTTAGCTTGTTTTTGAGCA
	Probe	[HEX]AGAAAAAGGCACGGTTGAGA[BHQ1]
erm(C)	Forward	TTGTTGATCACGATAATTTCCAA
	Reverse	CAAACCCGTATTCCACGATT
	Probe	[ROX]TGCAGTTTAAATTTCCTAAAAACCA[BHQ2]
